# The Effect of Epipharyngeal Abrasive Therapy (EAT) on the Baroreceptor Reflex (BR)

**DOI:** 10.7759/cureus.45080

**Published:** 2023-09-12

**Authors:** Ito Hirobumi

**Affiliations:** 1 Otolaryngology, Ito ENT Clinic, Funabashi, JPN

**Keywords:** inhibition of blood pressure variability, baroreceptor reflex (br), inhibition of parasympathetic nerve activity, active standing test (as test), heart rate variability analysis, temporal effects, cardiocirculatory autonomic activity, epipharyngeal abrasive therapy (eat), autonomic nervous system dysfunction, chronic epipharyngitis

## Abstract

Introduction

Epipharyngeal Abrasive Therapy (EAT) has been used as a treatment for chronic epipharyngitis, and although autonomic nerve stimulation has been pointed out as one of the mechanisms by which EAT produces therapeutic effects, there have been few reports examining this mechanism of action. This study investigated the effects of repeated EAT on autonomic nervous system activity in chronic epipharyngitis patients over time, using heart rate variability analysis. In addition, we conducted a loading test using the active standing test (AS test) to examine the effects of EAT on the baroreceptor reflex (BR).

Subjects and methods

A retrospective study was conducted on 39 patients who visited our clinic between July 2017 and November 2019 and underwent autonomic function tests with a diagnosis of chronic nasopharyngeal inflammation. The subjects were divided into two groups: the improvement group and the invariant group for comparison. Electrocardiographic recordings and blood pressure measurements were made under the stress of the AS test. Heart rate, high-frequency (HF) component, low-frequency (LF) component, and Coefficient of Variation on R-R interval were evaluated as indices of autonomic function. Component coefficient of variance high frequency was used as an index of parasympathetic function. ccvLF/ccvHF ratio was calculated by dividing the component coefficient of variance low frequency by ccvHF. The AS test was conducted in phase 1 in the initial resting sitting position, in phase 2 in the standing position, in phase 3 in the standing and holding the standing position, and in phase 4 in the seated and holding the sitting position. Systolic blood pressure, mean arterial pressure, and diastolic blood pressure were obtained in each phase. A paired t-test was used to compare the improved and invariant groups before and after treatment. The post-treatment comparison between the improved group and the invariant group was performed by unpaired t-test. Variation of the evaluation index over time was evaluated by repeated measures ANOVA. Multiple comparisons were corrected by the Bonferroni method.

Results

The EAT showed that parasympathetic activity was significantly suppressed in the improvement group, while the AS test showed significant fluctuations over time for the improvement and invariant groups. The interaction between the time course and the two factors in the improvement and invariant groups was not statistically evident. Although no significant difference was found, the improvement group showed a tendency to suppress parasympathetic activity and a tendency to stimulate sympathetic activity compared to the invariant group. Blood pressure in the improvement group showed a tendency to decrease.

Conclusions

EAT was found to suppress parasympathetic activity over time, and the AS test did not reveal an interaction effect of EAT on BR. However, there was a trend toward suppression of parasympathetic activity and stimulation of sympathetic activity in the improved group compared to the invariant group. Blood pressure in the improved group tended to decrease. It is possible that EAT may have a positive effect on autonomic neuropathy symptoms such as orthostatic dysregulation (OD), postural orthostatic tachycardia syndrome (POTS), etc. by stimulating the BRs. It is thought that the autonomic nervous system stimulating action and the immune system stimulating action act synergistically to express the therapeutic effect of EAT.

## Introduction

Epipharyngeal Abrasive Therapy (EAT) is used as a treatment for chronic epipharyngitis [1], and the following mechanisms of therapeutic effects of EAT have been pointed out: (1) astringent and bactericidal effects of zinc chloride, (2) purgative effects, and (3) vagus nerve stimulation effects [2]. The mechanism of action on the autonomic nervous system is thought to be stimulation of the autonomic nervous system by stimulating the vagus nerve projecting to the epipharynx and anti-inflammatory action via the inflammatory reflex caused by vagus nerve stimulation [3].

EAT has been reported to have therapeutic effects on autonomic dysfunction (AD), including orthostatic dysregulation (OD) [3], postural orthostatic tachycardia syndrome (POTS) [3], myalgic encephalomyelitis/chronic fatigue syndrome (ME/CFS) [4,5], long COVID (LC) [6-8], and other autonomic dysfunction. From these reports, it can be inferred that EAT influences cardiocirculatory autonomic nervous system activity. To date, however, few reports have examined the effects of EAT on cardiocirculatory autonomic nervous system activity.

Harada S reported the effect of epipharyngeal stimulation on the vasomotor reflex. He reported that EAT normalizes the vasomotor reflex [9]. Katori S performed the Mecholyl Test and analyzed the ear lobe vascular volume pulses. He reported that autonomic symptoms caused by chronic epipharyngitis are due to latent persistent stimulation and that EAT improved autonomic symptoms [10]. The author performed heart rate variability analysis of EAT autonomic reflexes and found that sympathetic and parasympathetic activities were alternately stimulated, and reported that the opposing excitatory and inhibitory stimulus inputs to the sympathetic and parasympathetic nervous systems by EAT may activate the autonomic reflex function [11]. However, the mechanism of action by which EAT affects cardiocirculatory autonomic nervous system activity is still largely unexplored.

This study used heart rate variability analysis to analyze the effects of repeated EAT on cardiocirculatory autonomic nervous system activity over time in patients with chronic epipharyngitis. The effects of EAT on baroreceptor reflex (BR) were also examined using an active standing test (AS test) [12]. We hypothesized that EAT improves AD in chronic epipharyngitis by activating the BR and that clarifying the effects of EAT on autonomic nervous activity in the cardiocirculatory system will be useful in clarifying the mechanism of therapeutic effects of EAT. The results of this study are presented in this report.

## Materials and methods

From July 2017 to November 2019, 1173 patients were diagnosed with chronic epipharyngeal inflammation according to Tanaka's diagnostic criteria [13] by visiting our clinic for EAT with the main complaints of posterior rhinorrhea, hoarseness, abnormal feeling, chronic fatigue, and so on. Among them, 39 patients underwent autonomic function testing using the AS test before and after the start of treatment. The medical records of these 39 patients were examined for gender, age, duration of illness, duration of treatment, number of treatments, changes in subjective symptoms, changes in objective findings, and changes in laboratory records. The total number of subjects was 39, with an average age of 42.8 years; eight males, with an average age of 44.5 years; and 31 females, with an average age of 42.4 years. Based on the results of the treatment efficacy evaluation, the patients whose symptoms improved were classified as the improvement group (target group), and the patients whose symptoms remained invariant were classified as the invariant group (control group), and the two groups were compared.

This study was a retrospective observational study based on existing medical records without obtaining new samples or information. This study was conducted in compliance with the "Declaration of Helsinki" and the "Ethical Guidelines for Medical Research Involving Human Subjects" (partially amended on March 10, 2022). Oral and written informed consent was obtained from all patients. When handling data and other materials related to the study, I made sure to protect the confidentiality of the subjects and not to include any information that could identify them in the publication of the study results. This study was conducted with the approval of the Ethics Review Committee of the Chiba Prefecture Health Physicians Association (approval No. 20210601008).

Diagnosis and treatment of chronic epipharyngitis and evaluation of other findings were performed using a band-limited optical endoscopy system (Pentax EPK-i7000 video processor, VNL11-J10 videoscope with a 3.5 mm dia. tip outer diameter; manufactured by PENTAX Corporation, Tokyo, Japan). The standard EAT procedure performed in this study was as follows. First, the patient was anesthetized with 1% Xylocaine to prevent pain in the nasal cavity before EAT, and hyperemia and swelling of the nasal mucosa were removed with a 0.01% adrenaline solution. Next, an endoscope was inserted nasally for endoscopic diagnosis. Next, while observing the nasopharynx, a Rouze swab soaked in 1% zinc chloride solution was used to perform EAT nasally. EAT was then performed orally using a Zermach pharyngeal crimp cotton swab impregnated with 1% zinc chloride solution. After observing the bleeding, the endoscope was removed, and the procedure was terminated.

Electrocardiograms (ECG) were derived using II induction with electrodes attached to the chest. ECG recordings were made using a heart rate monitor (Memory Heart Rate Monitor LRR-03 manufactured by ARM Electronics, Tokyo, Japan). The blood pressure monitor was a biometric monitor (Circlemates, A&D Corporation, Tokyo). The ECG detected the R-wave peak interval at a sampling frequency of 1000 Hz, and heart rate-linked continuous analysis was performed using the maximum entropy method [14]. Heart rate variability analysis was performed using heart rate variability analysis software (Crosswell Kitsu Meijin, Yokohama, Japan).

Heart rate (HR), high frequency (HF), low frequency (LF), and autonomic nervous activity (Coefficient of Variation on R-R interval; CVRR) were calculated as indices to evaluate autonomic nervous function. HF ranged from 0.15 to 0.40 Hz, and LF ranged from 0.04 to 0.15 Hz. CVRR is an aggregate of HF, LF, etc., and was used as a measure of the sum of autonomic nervous activity. The component coefficient of variance high frequency (ccvHF) is a fluctuation coefficient defined by Hayano and colleagues [15]. The ccvHF was used as an index of parasympathetic function; the HF and LF components reflect sympathetic and parasympathetic functions. The ccvLF/ccvHF ratio (ccvL/H), which is the component coefficient of variance low frequency (ccvLF) divided by ccvHF, was used as an index of sympathetic function [16].

For the AS test [17], the patient was first held in a resting sitting position for two minutes and then actively stood up. After standing up, the standing position was held for two minutes. Next, the patient was seated and held in the sitting position for one minute during the AS period, and ECG and blood pressure variability were continuously recorded [16]. The recording interval was divided into four phases: phase 1 for the resting sitting position, phase 2 for the standing position, phase 3 for the standing position, and phase 4 for the seated sitting position. The mean values of HR, CVRR, ccvHF, and ccvL/H in phase 1, HR in phase 2, and ccvHF in phase 4 were calculated. Systolic blood pressure (SBP), mean arterial pressure (MAP), and diastolic blood pressure (DBP) were determined in each phase.

The first AS test was performed and measured before the first EAT, and the second AS test was measured before the EAT after about 10 or more EAT treatments over a period of about 1 month. The results of the first and second AS test measurements were compared to examine the effect of EAT on BR. 39 patients who performed the first AS test were subjected to the Smirnov-Grubbs test, and one case was excluded as an outlier. Thirty-eight patients were subjected to the phase 1 measurements, The Kolmogorov-Smirnov test was used to confirm that the measures of autonomic function were normally distributed.

The degree of improvement of subjective symptoms at the second AS test evaluation was evaluated on a 3-point scale (improved, unchanged, or worsened), and the degree of improvement of objective findings at the second AS test evaluation was evaluated on a 3-point scale (improved, unchanged, or worsened). Based on the results of the medical record survey, patients in whom both subjective symptoms and objective findings improved were designated as the improvement group (target group). Cases in which subjective symptoms or objective findings remained unchanged or worsened were designated as the invariant group (control group). A comparison of the two groups was performed.

For the statistical study, the basic attributes of sex, age, and disease duration of the subjects were first determined; the mean duration of treatment and the mean number of treatments before the second AS test were determined.

Next, the effect of EAT on the AS test was compared between the two groups of patients: the improved group and the invariant group for pre- and post-treatment measures of HR, CVRR, ccv HF, and ccv L/H in phase 1, HR in phase 2, and ccv HF in phase 4, with statistical analysis using a The statistical analysis was performed by paired t-test.

Next, the effect of EAT on the AS test was compared between the two groups of post-treatment measurements: HR, CVRR, ccv HF, and ccv L/H in phase 1, HR in phase 2, and ccv HF in phase 4, with statistical analysis performed by unpaired t-test. unpaired t-test.

Two AS tests were performed, and 35 cases were able to record changes over time in all four phases. These 35 cases were analyzed. Within-group comparisons of pre- and posttreatment HR, CVRR, ccv HF, ccv L/H, SBP, MAP, and DBP measurements were made for the improved and invariant groups by repeated measures ANOVA (repeated measures (rm) ANOVA). Multiple comparisons were corrected by the Bonferroni method. The effect of EAT on measurements during the AS test was also examined.

The statistical software EZR version 2.6-2 (www.jichi.ac.jp/saitama-sct/SaitamaHP.files/statmed.html) was used for statistical analysis. A difference of less than 5% risk rate was considered statistically significant. No statistical sample size calculation was performed. With a sample size of 15 cases in the improvement group and 20 cases in the invariant group, the post hoc power was 41%, assuming a common standard deviation of 40% to detect a 20% difference in mean change from phase 1 to phase 2 in ccv HF between the improvement and invariant groups.

## Results

As a result of the evaluation, there were 16 cases in the improved group and 22 cases in the unchanged group. Table 1 shows the basic attributes of the subjects. Most of the subjects with chronic epipharyngitis were women in their 40s. The male-to-female ratio in the improved group was 1:2.2. The male-to-female ratio in the invariant group was 1 to 6.3. The average duration of treatment for both groups was about 2 months, and the average number of treatments was about 10. Subjective symptoms improved in 26 patients and remained invariant in 12 patients, for an improvement rate of 68.4%. The rate of improvement in other findings was 60.5%: 23 patients improved, 14 patients remained invariant, and one patient experienced an exacerbation of symptoms. Both subjective symptoms and objective findings improved in 16 patients, for an improvement rate of 42.1%.

**Table 1:** Basic statistics of 38 cases There were 16 cases in the improved group and 22 cases in the unchanged group. The numbers shown in the table indicate mean values. Numbers in parentheses indicate standard deviation values.

Basic Attributes of the Subject	Improvement group	Invariant group
Number of patients (Male)	16 (5)	22 (3)
Age ( year)	47.6 (14.9)	40.1 (13.6)
During the disease (month)	12.1 (13.1)	15.2 (24.1)
Length of treatment (month)	2.3 (1.1)	2.0 (0.8)
Number of treatments (times)	10.7 (5.0)	9.9 (6.2)

**Figure 1:**
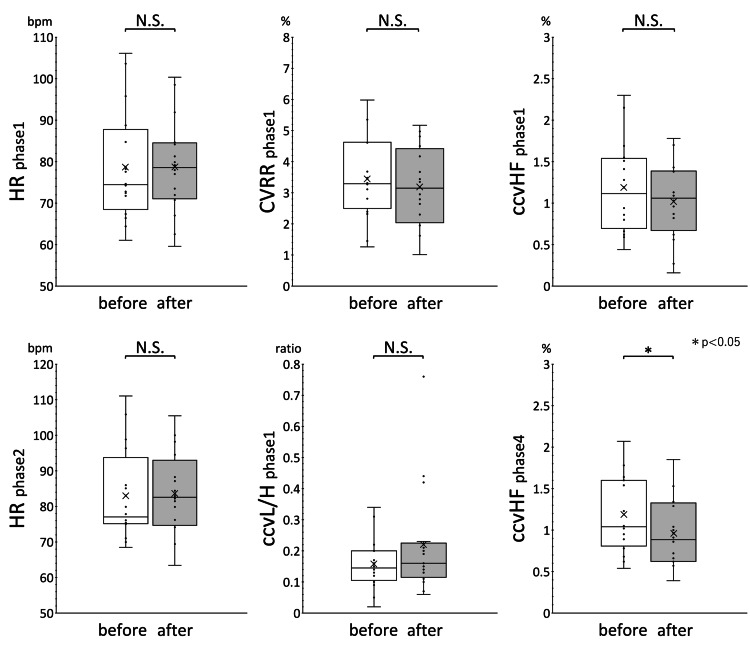
Within-group comparison of autonomic function indices before and after EAT treatment in the improvement group The results of the within-group comparison of HR, CVRR, ccvHF, and ccvL/H before and after treatment in phase 1, HR in phase 2, and ccvHF in phase 4 of the improvement group are shown. Data distribution is shown in box-and-whisker plots. The bottom of the whiskers indicate the minimum value, the bottom box indicates the first quartile (25th percentile), the middle line indicates the second quartile (median), the top box indicates the third quartile (75th percentile), and the top of the whiskers indicates the maximum value. The horizontal line in the box indicates the median and the mean is indicated by x. ccvHF showed a significant decrease after EAT treatment in phase 4. No significant differences were observed for the other evaluated indices. The median and mean of HR and ccvL/H both showed an increasing trend after EAT treatment. CVRR and ccvHF both showed a decreasing trend after EAT treatment.

**Figure 2:**
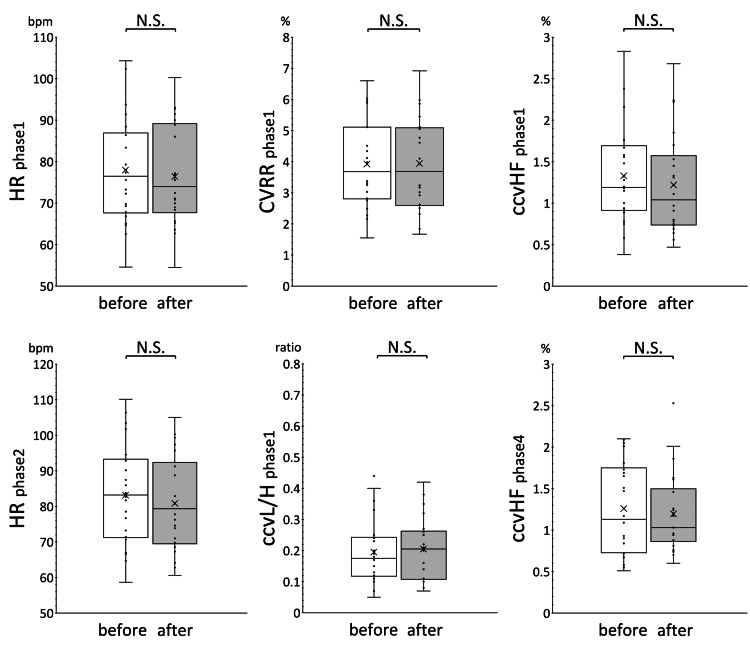
Within-group comparison of autonomic function indices before and after EAT treatment in the invariant group The results of the within-group comparison of HR, CVRR, ccvHF, and ccvL/H before and after treatment in phase 1, HR in phase 2, and ccvHF in phase 4 in the invariant group are shown. The indexes did not change significantly. Median and mean ccvL/H tended to increase after EAT treatment; median and mean HR and ccvHF tended to decrease after EAT treatment; median and mean CCVR did not change.

Figure 1 shows the results of within-group comparison of HR, CVRR, ccvHF, and ccvL/H before and after treatment in phase 1, HR in phase 2, and ccvHF in phase 4 in the improvement group. Figure 2 shows the results for the invariant group. The results of the within-group comparison show that EAT treatment significantly reduced ccvHF in phase 4 of the improvement group. No significant changes were observed in the other parameters. HR and ccvL/H in the improvement group showed an increasing trend, while CVRR and ccvHF showed a decreasing trend, although there were no significant differences. The trend of variation in each measure in the invariant group was not clear.

**Figure 3:**
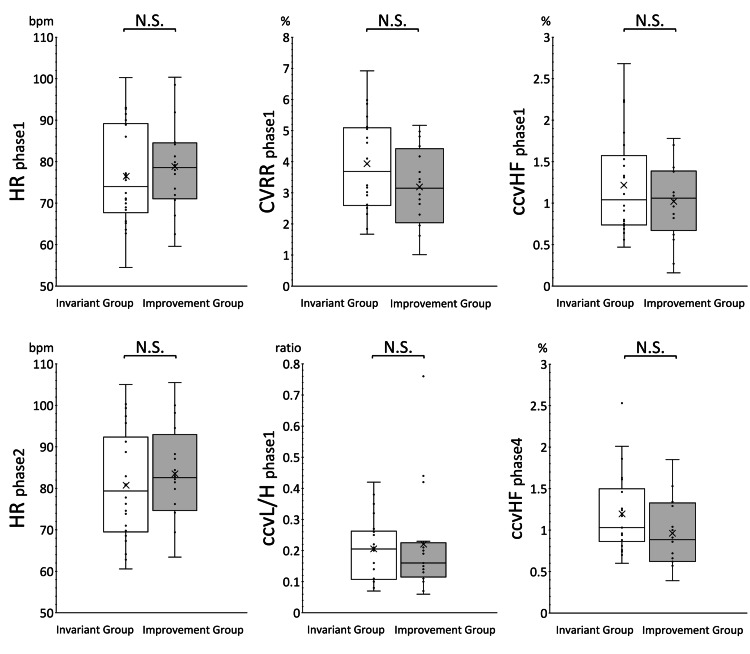
Comparison of autonomic function indices after EAT treatment between the improvement group and invariant group The comparison of HR, CVRR, ccvHF, and ccvL/H in phase 1, HR in phase 2, and ccvHF in phase 4 after EAT treatment is shown in white and gray box whiskers, respectively. There were no significant differences in autonomic indexes; median and mean HR tended to increase after EAT treatment; median and mean CVRR and ccvHF tended to decrease after EAT treatment; no change in ccvL/H was evident; and median and mean ccvL/H tended to increase after EAT treatment.

Figure 3 shows the results of the post-treatment comparison of HR, CVRR, ccvHF, and ccvL/H in phase 1, HR in phase 2, and ccvHF in phase 4 for the improvement group and the invariant group. The post-treatment comparison between groups showed no significant differences in any of the measures.

**Figure 4:**
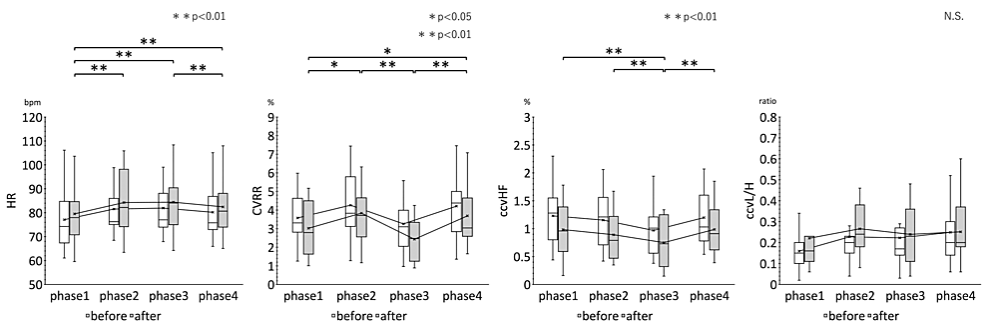
Results of multiple comparisons of HR, CVRR, ccvHF, and ccvL/H in the improvement group Changes over time in HR, CVRR, ccvHF, and L/H measurements in the four phases are shown. A comparison of changes over time before and after EAT treatment in 15 patients was performed by rm ANOVA (Bonferroni correction). However, the interaction effect of EAT treatment was not clear. There was a trend toward suppression of each index in each phase after EAT treatment compared to pre-treatment. HR: Heart rate; CVRR: Coefficient of Variation on R-R interval; ccvHF: Component coefficient of variance high frequency; ccvLF: Component coefficient of variance low frequency; ccvL/H: ccvLF/ccvHF ratio * P <0.05, ** P<0.01

**Figure 5:**
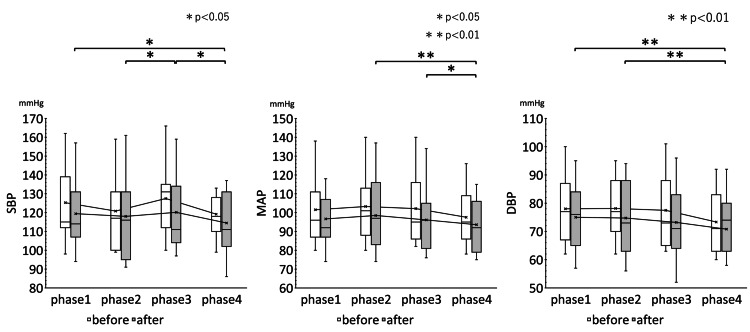
Results of multiple comparisons of SBP, MAP, and DBP in the improvement group Changes in SBP, MAP, and DBP over time in the four phases are shown. The white box whiskers before EAT treatment and the gray box whiskers after EAT treatment show significant changes in SBP, MAP, and DBP, respectively, but the interaction effect of EAT treatment was not evident. There was a trend toward suppression of each index in each phase after EAT treatment compared to before treatment. * P<0.05, ** P<0.01 SBP: Systolic blood pressure; MAP: Mean arterial pressure; DBP: Diastolic blood pressure; EAT: Epipharyngeal abrasive therapy

The rm ANOVA results showed that the pre-and post-treatment HR, CVRR, ccvHF, ccvL/H, SBP, MAP, CVRR, ccvHF, ccvL/H and analysis of variance of the mean values of HR, CVRR, ccvHF, ccvL/H, SBP, MAP, and DBP before and after treatment in the four phases of the improvement group are shown in Table 2. The results of multiple comparisons of HR, CVRR, ccvHF, and ccvL/H in the improvement group are shown in Figure 4. Figure 5 shows the results of multiple comparisons of SBP, MAP, and DBP in the improvement group.

**Table 2:** Changes over time and results of analysis of variance of mean values of HR, CVRR, ccvHF, ccvL/H, SBP, MAP, and DBP before and after treatment in the four phases of the improvement group The numbers shown in the table indicate mean values. Numbers in parentheses indicate standard deviation values. * P < 0.05, ** P < 0.01 HR: Heart rate; CVRR: Coefficient of Variation on R-R interval; ccvHF: Component coefficient of variance high frequency; ccvLF: Component coefficient of variance low frequency; ccvL/H: ccvLF/ccvHF ratio; SBP: Systolic blood pressure; MAP: Mean arterial pressure; DBP: Diastolic blood pressure

improvement group		phase 1	phase 2	phase 3	phase 4	Main Effects		Interaction	
						F value	P value	F value	P value
HR	before	77.09 (11.83)	81.52 (11.62)	81.95 (11.98)	80.19 (11.47)	33.36	0.00**	0.67	0.97
	after	79.52 (12.78)	84.26 (12.80)	84.42 (12.64)	82.45 (12.58)				
CVRR	before	3.58 (1.21)	4.26 (1.78)	3.26 (1.52)	4.21 (1.57)	15.13	0.00**	0.34	0.79
	after	3.02 (1.35)	3.83 (1.90)	2.43 (1.09)	3.69 (1.46)				
ccvHF	before	1.23 (0.53)	1.53 (0.45)	0.97 (0.41)	1.20 (0.46)	9.81	0.00**	0.11	0.95
	after	0.98 (0.48)	0.89 (0.42)	0.75 (0.41)	0.99 (0.40)				
ccvL/H	before	0.16 (0.08)	0.23 (0.13)	0.22 (0.15)	0.25 (0.14)	4.27	0.01*	0.93	0.43
	after	0.22 (0.18)	0.27 (0.12)	0.24 (0.12)	0.25 (0.14)				
SBP	before	125.26 (18.50)	120.80 (18.04)	127.47 (17.72)	119.13 (14.06)	5.60	0.00**	0.53	0.66
	after	119.40 (16.32)	118.00 (16.32)	120.13 (18.31)	114.47 (14.75)				
MAP	before	101.53 (16.88)	103.27 (17.05)	102.13 (17.06)	97.53 (14.14)	6.13	0.00**	0.22	0.88
	after	96.67 (15.70)	98.47 (19.44)	96.13 (16.12)	93.60 (12.63)				
DBP	before	78.00 (10.65)	78.13 (10.43)	77.47 (11.80)	73.33 (10.41)	8.11	0.00**	0.25	0.86
	after	75.00 (10.79)	74.73 (12.04)	73.20 (12.07)	70.80 (13.66)				

**Figure 6:**
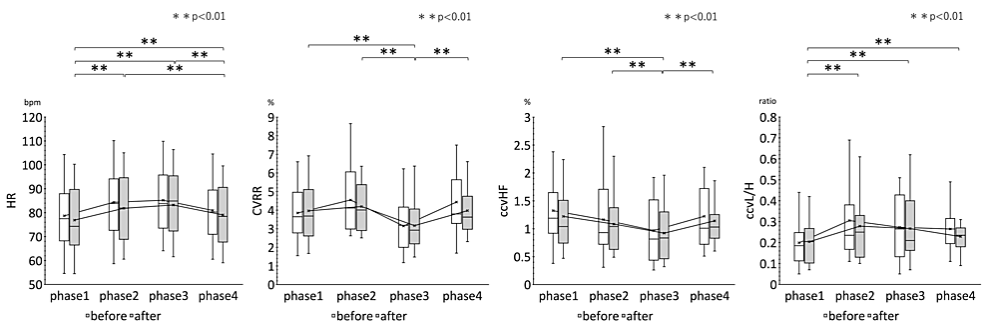
Results of multiple comparisons of HR, CVRR, ccvHF, and ccvL/H in the invariant group Changes over time in HR, CVRR, ccvHF, and ccvL/H measurements in the four phases are shown. The changes in HR, CVRR, ccvHF, and ccvL/H before and after EAT treatment are shown in white box whiskers before EAT treatment and gray box whiskers after EAT treatment. rm ANOVA ( Bonferroni correction ) was used to compare the changes over time before and after EAT treatment in 20 patients. The interaction effect of EAT treatment was not clear. The change in each index after EAT treatment was not evident compared to that before treatment. ** P<0.01 HR: Heart rate; CVRR: Coefficient of Variation on R-R interval; ccvHF: Component coefficient of variance high frequency; ccvLF: Component coefficient of variance low frequency; ccvL/H: ccvLF/ccvHF ratio; EAT: Epipharyngeal abrasive therapy

**Figure 7:**
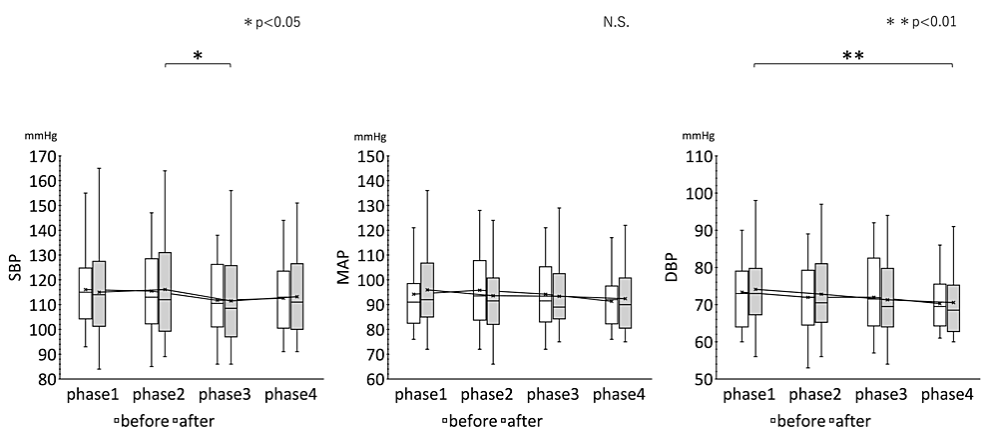
Results of multiple comparisons of SBP, MAP, and DBP in the invariant group Changes in SBP, MAP, and DBP over time in the four phases are shown. The interaction effect of EAT treatment was not evident. Changes in each of the assessed indices after EAT treatment were not evident. * P<0.05, ** P<0.01 SBP: Systolic blood pressure; MAP: Mean arterial pressure; DBP: Diastolic blood pressure; EAT: Epipharyngeal abrasive therapy

The results of rm ANOVA and analysis of variance for the mean changes over time of HR, CVRR, ccvHF, ccvL/H, SBP, MAP, and DBP before and after treatment in the four phases of the invariant group are shown in Table 3. The results of multiple comparisons of HR, CVRR, ccvHF, and ccvL/H for the unchanged group are shown in Figure 6. Figure 7 shows the results of multiple comparisons of SBP, MAP, and DBP in the improvement group.

**Table 3:** Changes over time in the mean values of HR, CVRR, ccvHF, ccvL/H, SBP, MAP, and DBP before and after treatment in the four phases of the invariant group and results of analysis of variance The numbers shown in the table indicate mean values. Numbers in parentheses indicate standard deviation values. * P<0.05, ** P<0.01 HR: Heart rate; CVRR: Coefficient of Variation on R-R interval; ccvHF: Component coefficient of variance high frequency; ccvLF: Component coefficient of variance low frequency; ccvL/H: ccvLF/ccvHF ratio; SBP: Systolic blood pressure; MAP: Mean arterial pressure; DBP: Diastolic blood pressure

invariant group		phase 1	phase 2	phase 3	phase 4	Main Effects		Interaction	
						F value	P value	F value	P value
HR	before	78.69 (12.96)	84.35 (13.96)	85.20 (13.98)	80.95 (11.96)	39.63	0.00**	0.12	0.95
	after	76.88 (12.27)	81.84 (13.46)	83.21 (13.49)	79.07 (12.37)				
CVRR	before	3.85 (1.36)	4.55 (1.77)	3.15 (1.33)	4.44 (1.63)	18.22	0.00**	1.23	0.30
	after	3.96 (1.49)	4.20 (1.29)	3.17 (1.19)	3.98 (1.29)				
ccvHF	before	1.32 (0.60)	1.16 (0.62)	0.97 (0.54)	1.22 (0.54)	13.38	0.00**	0.10	0.96
	after	1.22 (0.60)	1.08 (0.48)	0.92 (0.46)	1.14 (0.44)				
ccvL/H	before	0.20 (0.11)	0.31 (0.18)	0.27 (0.15)	0.27 (0.10)	7.97	0.00**	0.48	0.68
	after	0.20 (0.10)	0.28 (0.15)	0.27 (0.14)	0.23 (0.06)				
SBP	before	116.05 (16.17)	115.45 (16.45)	111.70 (15.43)	112.55 (14.18)	3.32	0.02*	0.13	0.94
	after	115.00 (18.68)	116.10 (19.60)	111.50 (17.62)	113.20 (15.20)				
MAP	before	94.20 (14.64)	95.80 (15.67)	94.20 (13.80)	91.30 (10.71)	2.91	0.04*	1.15	0.33
	after	95.95 (15.03)	93.60 (16.68)	93.35 (14.42)	92.40 (12.45)				
DBP	before	73.35 (8.84)	71.95 (9.45)	72.00 (9.95)	70.25 (7.12)	6.80	0.00**	0.44	0.73
	after	74.10 (10.48)	72.80 (10.89)	71.30 (10.67)	70.55 (9.07)				

 

The mean values of each autonomic evaluation index in the improvement group and the invariant group fluctuated significantly over time. In other words, a main effect of time course was observed. The interaction between time course and the two factors in the improvement and invariant groups was not statistically significant. In other words, there was no clear difference in the effect of EAT on the improvement and invariance groups.

The results of multiple comparisons showed that both the improvement group and the invariant group showed significant fluctuations in each of the assessment indices over time. HR in the improvement group and the invariant group increased in phases 2 and 3, and then returned to the same state as in phase 1 in phase 4. ccvHF showed an opposite trend to HR. ccvHF decreased in phases 2 and 3, and then returned to the same state as in phase 1 in phase 4. ccvHF increased in phase 2, and then decreased in phase 3, and returned to the same state as in phase 1 in phase 4. CVRR increased in phase 2, decreased in phase 3, and increased again in phase 4. ccvL/H showed a tendency to increase in phase 2, and remained at the same value in phase 3 and phase 4. The mean and median fluctuations of the second measurement in the improvement group were larger than those in the invariant group. In the improvement group, CVRR and ccvHF decreased, while HR and ccvL/H increased. The second SBP, MAP, and DBP measurements of the improvement group were lower than the first. The second blood pressure fluctuations in the improvement group tended to be suppressed. In the invariant group, the variation between the first and second measurements was not evident. The effect of EAT on blood pressure variability was not statistically clear, but the improvement group showed a greater effect of EAT on blood pressure variability than the invariant group.

Table 4 shows the results of the analysis of variance and change in mean values over time for the improvement group and the invariant group after treatment. Compared to the invariant group, the improvement group showed a decreasing trend in CVRR and ccvHF over time, and an increasing trend in HR and ccvL/H. The improvement group showed a significant change in MAP and DBP over time, and the invariant group showed a significant change in SBP over time. MAP and DBP showed significant changes over time, but SBP did not show significant changes. The interaction between the time course and the two factors in the improvement and invariant groups was not clear. In other words, the post-treatment group comparisons were not clear regarding the effect of EAT on each of the measures.

**Table 4:** Changes over time in mean values of HR, CVRR, ccvHF, ccvL/H, SBP, MAP, and DBP and results of analysis of variance by group comparison between improvement group and invariant group after treatment The numbers shown in the table indicate mean values. Numbers in parentheses indicate standard deviation values. * P<0.05, ** P<0.01 HR: Heart rate; CVRR: Coefficient of Variation on R-R interval; ccvHF: Component coefficient of variance high frequency; ccvLF: Component coefficient of variance low frequency; ccvL/H: ccvLF/ccvHF ratio; SBP: Systolic blood pressure; MAP: Mean arterial pressure; DBP: Diastolic blood pressure

Group Comparison		phase 1	phase 2	phase 3	phase 4	Main Effects		Interaction	
						F value	P value	F value	P value
HR	improvement group	79.52 (12.78)	84.26 (12.80)	84.42 (12.64)	82.45 (12.58)	27.83	0.00**	0.89	0.45
	invariant group	76.88 (12.27)	81.84 (13.46)	83.21 (13.49)	79.07 (12.37)				
CVRR	improvement group	3.02 (1.35)	3.83 (1.90)	2.43 (1.09)	3.69 (1.46)	17.00	0.00**	1.43	0.24
	invariant group	3.96 (1.49)	4.20 (1.29)	3.17 (1.19)	3.98 (1.29)				
ccvHF	improvement group	0.98 (0.48)	0.89 (0.42)	0.75 (0.41)	0.99 (0.40)	9.20	0.00**	0.22	0.86
	invariant group	1.22 (0.60)	1.08 (0.48)	0.92 (0.46)	1.14 (0.44)				
ccvL/H	improvement group	0.22 (0.18)	0.27 (0.12)	0.24 (0.12)	0.25 (0.14)	3.46	0.02*	0.78	0.51
	invariant group	0.20 (0.10)	0.28 (0.15)	0.27 (0.14)	0.23 (0.06)				
SBP	improvement group	119.40 (16.32)	118.00 (16.32)	120.13 (18.31)	114.47 (14.75)	1.62	0.19	1.87	0.14
	invariant group	115.00 (18.68)	116.10 (19.60)	111.50 (17.62)	113.20 (15.20)				
MAP	improvement group	96.67 (15.70)	98.47 (19.44)	96.13 (16.12)	93.60 (12.63)	2.91	0.04*	1.11	0.35
	invariant group	95.95 (15.03)	93.60 (16.68)	93.35 (14.42)	92.40 (12.45)				
DBP	improvement group	75.00 (10.79)	74.73 (12.04)	73.20 (12.07)	70.80 (13.66)	6.15	0.00**	0.35	0.79
	invariant group	74.10 (10.48)	72.80 (10.89)	71.30 (10.67)	70.55 (9.07)				

## Discussion

The purpose of this study was to clarify the effects of EAT on the autonomic nervous system activity of the cardiocirculatory system over time using heart rate variability analysis, and statistical analysis was performed on the changes in autonomic function assessment indices in the improvement group and the invariant group. The novelty of this study was the evaluation of changes in autonomic activity in chronic epipharyngitis before and after treatment, separately for parasympathetic and sympathetic activity. The possibility of EAT influencing changes in autonomic activity over time was evaluated. The subjects were divided into two groups, an improvement group, and an invariant group, and a comparative evaluation of the changes in autonomic nervous system activity in the two groups was conducted after the AS test was administered. Within-group comparisons between the improvement group and the invariant group showed significant changes in autonomic function as a result of AS test loading, respectively. Post-treatment comparison between the improvement and invariant groups showed no significant difference between the improvement and invariant groups. However, the post-treatment improvement group showed greater fluctuations in autonomic evaluation indices than the invariant group, Parasympathetic activity was suppressed, sympathetic activity was stimulated, and blood pressure tended to decrease. The post-treatment changes in the improvement group were thought to be due to changes in BR, suggesting that EAT treatment may have an effect on BR.

This study rated the rate of improvement on a three-point scale. The improvement rate of subjective symptoms was 68.4%, and the improvement rate of objective findings was 60.5%. The cure rate for the improvement group, which showed improvement in both subjective symptoms and objective findings, was 42.1%. Ohno Y evaluated a group of patients who had undergone EAT approximately the same number of times and for the same length of time, using a 4-point scale. Ohno Y reported an improvement rate of 79.5% for the chief complaint and 87.7% for the local findings [18]. Compared to Ohno's paper, the improvement rate in this study was lower. This may be due to the more stringent criteria used in the evaluation methods of this study. It has been reported that chronic epipharyngitis tends to be more common in women [19]. The male-to-female ratio in the improvement group in this study was 1 to 2.2, while the male-to-female ratio in the unchanged group was 1 to 6.3, suggesting that women tend to have a harder time being cured than men. The average duration of treatment tended to be longer, and the average number of treatments tended to be higher in the improvement group, suggesting that increasing the duration and number of treatments may increase the cure rate.

The vasomotor reflex is influenced by the stiffness and elasticity of the vascular wall and consists of the vasoconstrictor reflex and the vasodilator reflex [20]. Harada analyzed the finger vasomotor reflex during a single EAT stimulation and reported that the immediate effect of EAT was to initially elicit the vasoconstrictor reflex and then the vasodilator reflex [21]. In the same paper, it was also reported that when symptoms are improved by EAT, the vasomotor reflex becomes smooth [21]. In the Harada paper, sympathetic activity was first stimulated and then parasympathetic activity was stimulated; when symptoms were improved by EAT, the vasomotor reflex became smooth, suggesting that EAT has the function of normalizing reflex activity. This study found that EAT suppressed parasympathetic activity over time in the improvement group. Relatively speaking, sympathetic activity is thought to be stimulated. The author reported that EAT has a stimulating effect on sympathetic nerve activity [22]. In this study, EAT seemed to facilitate autonomic reflex activity in the improvement group, suggesting that EAT improves autonomic reflex activity and expresses a therapeutic effect.

When sympathetic nerve activity is stimulated by anxiety, fear, or pain stimuli, blood pressure rises. Parasympathetic activity is increased to decrease this elevated blood pressure, triggering the vasovagal reflex (VV reflex), resulting in hypotension and bradycardia. Excessive vasovagal reflexes may result in loss of consciousness or cardiac arrest [23]. Pathological vagal reflexes are more likely to be elicited in ME/CFS because vagal homeostasis is impaired [5]. EAT is often painful, but empirically, vasovagal reflex syncope is rarely associated. The inhibitory effect of EAT on parasympathetic activity may suppress the development of the pathological vasovagal reflex. Based on the results of this study, the suppression of parasympathetic activity by EAT may result in an improvement in sympathetic reflex activity, thereby ameliorating cardiocirculatory autonomic symptoms.

In this study, a comparison between groups was made for the improvement group and the invariant group after treatment, but no significant difference was found in the autonomic evaluation indices. The fact that there was no difference between the improvement group and the invariant group in the autonomic evaluation index by the second AS test suggests that the invariant group may have experienced the same autonomic stimulating effect of EAT as the improvement group. In this study, the improvement group was defined as those with improvement in both subjective symptoms and objective findings, while those with improvement in either subjective symptoms or objective findings were classified as the invariant group. A time lag has been reported to exist between improvement of subjective symptoms and objective findings [19], and the EAT effect on LC has been reported to be preceded by improvement of objective findings rather than subjective symptoms [8]. It is possible that the method used in this study to classify patients into improved and invariant groups based on subjective symptoms and objective findings was not appropriate. In the future, it will be necessary to develop an evaluation index that can more easily and accurately evaluate subjective symptoms and objective findings.

It is also possible that the classification of the improvement group and the invariant group in this study did not significantly differ in the effectiveness of EAT treatment because the severity of chronic epipharyngitis and the severity of autonomic symptoms before the start of treatment were not evaluated. Kawada analyzed and reported on the static and dynamic characteristics of the arterial baroreceptor reflex. He pointed out the possibility that the autonomic regulatory system responds differently in normal and pathological conditions [24]. Future comparative evaluation based on classification by symptom and severity of illness may be necessary.

In this study, the effect of EAT over time was analyzed using rm ANOVA. Results showed that HR, CVRR, ccvHF, and ccvL/H in both improvement and invariant groups showed fluctuations over time in phase 2 and phase 3 compared to phase 1 and stabilized in phase 4. Blood pressure also showed fluctuations in phases 2 and 3 and stabilized in phase 4. The subject patients responded normally to BR, and the homeostasis that maintains blood pressure was considered to be preserved.

The results of this study showed a trend toward greater variability in autonomic assessment indices after EAT treatment in the improvement group compared to the invariant group. CVRR and ccvHF decreased in each phase; HR and L/H increased. The improvement group showed a tendency to suppress parasympathetic activity and stimulate sympathetic activity compared to the invariant group; SBP, MAP, and DBP decreased, and blood pressure variability showed a decreasing trend. This may be due to the activation of BR by stimulating autonomic reflex activity and maintaining blood pressure homeostasis.

When a normal subject is subjected to a standing test, the BR acts to regulate blood pressure fluctuations and maintain blood pressure The mechanism of action of the BR is that the initial active attempt to stand up stimulates the sympathetic nervous system by information from the central command of the cerebral cortex. On the other hand, the gravitational effect of the standing load causes a retention of blood in the veins of the lower extremities and trunk. Cardiac output is reduced and blood pressure is lowered because blood returning to the heart from the lower extremity veins is reduced. In the normal state, the BRs act quickly to stimulate sympathetic activity and inhibit parasympathetic activity, thereby increasing heart rate and cardiac contractility, and blood pressure is maintained by vasoconstriction. Further standing increases circulating blood volume through activation of the renin-angiotensin-aldosterone system and secretion of antidiuretic hormone. Sympathetic activity is then suppressed and parasympathetic activity is stimulated, which stabilizes heart rate, cardiac contractility, and vasoconstriction to maintain blood pressure appropriate for standing [16]. CFS, and LC, autonomic neuropathy symptoms such as dizziness and dizziness may develop. In the improvement group in this study, the BR was activated by EAT, and blood pressure fluctuation was reduced, suggesting that homeostasis was working to maintain blood pressure and that the activation of the BR by EAT may have improved the cardiocirculatory autonomic neuropathy symptoms caused by chronic epipharyngitis.

Katori performed a methacholine chloride muscle injection (Mecholyl BAPG test) and pharmacological autonomic function tests while recording the auricular vascular volume pulse wave in patients with chronic epipharyngitis. A symptom classification of chronic epipharyngitis was performed and autonomic function was evaluated for each symptom. The dizziness type showing autonomic dysfunction symptoms was observed in most cases with decreased parasympathetic function and increased sympathetic function, but there were also cases with increased parasympathetic function and decreased sympathetic function. The study revealed that autonomic dysfunction due to chronic epipharyngitis is not uniform. Katori reported that when autonomic neuropathy symptoms are improved by EAT, autonomic function is also normalized [10]. In Katori's paper, the parasympathetic predominant cases may induce neurally mediated syncope [16], and EAT may suppress the pathological vasovagal reflex and normalize the parasympathetic function. Sympathetic function predominant cases may also induce POTS and other symptoms [16]. Excessive sympathetic hyperactivity induces the Reilly phenomenon [25], and EAT is thought to suppress excessive sympathetic reflexes and normalize sympathetic function. Since chronic epipharyngitis is characterized by a mixture of parasympathetic and sympathetic predominant cases, it is important to evaluate autonomic function prior to the start of treatment.

When afferent signals increase with chronic persistent stimulation due to chronic epipharyngitis, sympathetic nerve activity increases, and autonomic nerve activity, blood pressure, and other parameters also change. In such conditions, the regulatory system for autonomic nerve activity is also thought to be abnormal. Yamamoto reported that the correlation between heart rate and blood pressure is different in diseases such as acute cerebral infarction and myasthenic sclerosis, because the central autonomic nervous system is impaired differently in each disease [26]. EAT may have the function of regulating autonomic nervous system activity. Although the evaluation of autonomic function by heart rate variability analysis is considered to have limitations, this study suggests that EAT suppresses parasympathetic activity and stimulates sympathetic activity to produce therapeutic effects on cardiocirculatory autonomic dysfunction symptoms.

Next, a review of the literature on the mechanism of therapeutic effects of autonomic nerve stimulation of EAT is in order. Parasympathetic and sympathetic nerves are distributed from the posterior wall of the nasopharynx to the canal, and both parasympathetic and sympathetic fibers flow into the pterygopalatine ganglion (SPG). Trigeminal autonomic cephalalgias (TACs) [27] include cluster headache, SPG or vidian nerve irritation [28] and inflammatory reflex pain. These disorders are thought to stimulate the trigeminal nerve in the SPG region, inducing parasympathetic reflexes [29].

Parasympathetic reflex symptoms of TACs include mydriasis, eyelid drooping, lacrimal secretion, facial cutaneous vasodilation, and trunk arterial blood pressure fluctuations. Trigeminal nerve stimulation produces a hypotensive or elevated effect by input to the trigeminal spinal tract nucleus. Input to the middle subnucleus of the trigeminal spinal tract is involved in the vasodilatory response, and input to the caudal subnucleus of the trigeminal spinal tract is involved in the vasoconstrictor response. The conflicting cardiovascular responses show differences among stimulus conditions, animal species, and individuals [30]. Although much remains unexplored regarding the reflex pathways of the baroreflex, given the effects of EAT on cardiocirculatory autonomic activity, it is likely that EAT induces trigeminal sympathetic and trigeminal parasympathetic reflexes that influence vasomotor and supraventricular centers.

Christine reported that blocking SPG (Sphenopalatine Ganglion block: SPG-block) improves symptoms of TACs [31]. Hosseini et al. reported that SPG stimulation (Sphenopalatine Ganglion Stimulation: SPG-Stim) (1) enhances collateral blood circulation, (2) stabilizes the blood-brain barrier, (3) provides direct neuroprotection, and (4) enhances brain plasticity [32]. Cheyuo et al. reported that vagal afferents project to the nucleus tractus solitarius (NTS) and from the NTS to the anterior, middle, and posterior cerebral arteries and basilar artery to modulate cerebral blood flow in a NO-activated manner [33]. Since vagus nerve stimulation suppresses trigeminal parasympathetic reflexes [34], SPG electrical stimulation therapy (SPG-Stim), in which an electrical device is implanted in the SPG for symptomatic relief of cluster headaches, has been clinically applied [35]. SPG-Stim is thought to improve cerebral blood flow by stimulating vagal nerve activity, thereby expressing the effect of restoring brain function [36]. Tanaka reported Intranasal Sphenopalatine Ganglion Stimulation (INSPGS) [6]. INSPGS stimulates both sympathetic and parasympathetic nerves. INSPGS is thought to improve cerebral blood flow and exert its effects.

Vagus nerve stimulation enhances cranial plasticity (neural plasticity enhancement) and improves autonomic nervous system function [37]. when the mucosa near the epipharyngeal mucosa is abraded with EAT, afferent nerves in the SPG-dominated area are stimulated, and both sympathetic and parasympathetic nerves are stimulated to produce the effect The EAT is thought to stimulate both sympathetic and parasympathetic nerves. Although EAT expresses prompting and inhibitory effects by alternately stimulating both sympathetic and parasympathetic activities, it is possible that the effects produced may differ depending on the stimulus intensity, the site of stimulation, and the timing of stimulation.

Next, the interaction of EAT with the immune system and the autonomic nervous system will be discussed in the literature. Nishi et al. conducted an immunohistological study and reported that EAT suppresses interleukin (IL)-6 and TNFα production in the epipharyngeal mucosa [38]. EAT has an anti-inflammatory effect on the local epipharyngeal mucosa. Overproduction of IL-6, an inflammatory cytokine, is involved in the development of autoimmune and inflammatory diseases [39]. IL-6 has been reported to attenuate arterial baroreceptor reflex function in the medullary solitary bundle nucleus [40]. Therefore, it is likely that BR is attenuated in cases of chronic epipharyngitis, and EAT may improve BR by suppressing inflammatory cytokines.

Mogidate reported that EAT decreased fractional exhaled nitric oxide (FeNO) levels [41,42], and the decrease in FeNO may be due to the suppression of type 2 inflammation, which is regulated by type 2 innate lymphocytes. lymphocytes express β2 adrenergic receptors, and sympathetic stimulation of β2 adrenergic receptors suppresses type 2 inflammation [43]; sympathetic stimulation by EAT may suppress type 2 inflammation.

Tracey KJ reported an inflammatory reflex in which the immune system is regulated by stimulation of the nervous system. He found that vagus nerve stimulation suppressed the production of inflammatory cytokines [44]. Vagus Nerve Stimulation (VNS), an electrical stimulation of the afferent vagus nerve to enhance the function of inhibitory systems such as GABA, has been clinically applied to treat intractable epilepsy [45]. Transcutaneous VNS also enhances brain plasticity and has been clinically applied in stroke rehabilitation [46]. Auricular vagus nerve stimulation has also been reported to be useful for POTS [47]. Afferent vagus nerve stimulation may affect both the nervous system and the immune system; EAT may stimulate the vagus nerve to produce VNS-like effects.

Murakami reported that inflammation is induced in non-immune cells by IL-6 signals and named this mechanism of inflammation formation centered on non-immune cells the inflammation amplifier [48]. He found Gateway Reflex, in which sympathetic nerve stimulation activates β1 adrenergic receptors to induce inflammation and open the blood-brain barrier at specific vascular sites [49]. Murakami investigated the molecular mechanisms of the effects of pain stress, mental stress, electrical stimulation, and sleep disturbance on chronic inflammatory pathology, and found that activation of sensory and sympathetic nerves is an important factor in inflammatory activation in the central nervous system [50]. Adrenergic receptor-mediated responses are thought to vary depending on the receptor and the cells expressing it. Autonomic stimulation of the immune system may regulate the inflammatory response positively or negatively, depending on the receptor that receives the input. Based on the above, it is possible that chronic epipharyngitis induces inflammation in specific areas of the autonomic nerve center, and that EAT may regulate inflammation in specific areas of the autonomic nerve center.

Various symptoms such as fatigue, dyspnea, brain fog, and autonomic neuropathy develop in LC [51], and it has been reported that these may be due to vagus nerve damage [52]. Chronic irritation of the vagus nerve is thought to induce encephalitis, resulting in the development of autonomic neuropathy symptoms; The usefulness of INSPGS as a treatment for LC has been reported [6]. It has been reported that EAT for LC with chronic epipharyngitis tends to suppress parasympathetic function [8]. This study also revealed a suppressive effect of EAT on parasympathetic activity, suggesting that EAT may exert its therapeutic effect by suppressing the increase in parasympathetic activity caused by vagus nerve inflammation.

Kobori analyzed the reflex waves induced by trigeminal nerve stimulation and reported that trigeminal nerve stimulation stimulates the vagus nerve reflex [53]. Conditioning stimulation of the superior laryngeal nerve (vagus nerve) stimulates the laryngeal reflex (strangulation reflex) [54]. The preceding trigeminal and vagus nerve stimulation serves as the conditioning stimulus and controls the reflex [55]. In the standard EAT procedure, in which nasal stimulation is followed by oral stimulation, it is possible that the initial insertion of the fiberscope into the nasal cavity and swabbing may serve as the conditioned stimulus, prompting and controlling the following nasopharyngeal posterior wall rub stimulus. The standard EAT with nasal stimulation followed by oral stimulation is experienced to be more therapeutic than EAT with nasal stimulation alone or EAT with oral stimulation alone. This may be due not only to differences in the size of the area to be rubbed but also to differences in autonomic nerve stimulation effects; trigeminal and vagus nerve stimulation by EAT may interact to produce the effect.

Airway reflexes are a general term for various reflexes that are triggered by stimuli applied to the airways and occur in the respiratory and circulatory systems. In addition to airway defense reflexes, there are other important reflex mechanisms involved in respiratory control, swallowing control, and vocal control [56]. EAT is thought to exert its therapeutic effect by inducing various autonomic reflexes. The immune system and the autonomic nervous system interact with each other [57]. EAT is thought to exert its therapeutic effects not only through its anti-inflammatory effects on the local epipharyngeal mucosa but also by stimulating autonomic reflexes and the immune system through its sympathomimetic and parasympathomimetic effects. Even after local inflammatory findings have improved with EAT, continued EAT may improve autonomic symptoms in patients with residual subjective symptoms. It is possible that autonomic nerve stimulation by EAT activates the autonomic nerve center and enhances cranial nerve plasticity (neural plasticity enhancement)[37], improving autonomic nerve function and symptoms.

This paper examined the effects of EAT on autonomic nervous system stimulation on cardiocirculatory autonomic activity. It was found that parasympathetic activity was suppressed in the improvement group. The mechanism by which this effect is mediated by the therapeutic effect is discussed. The results of this study and the literature review indicate that the EAT affects the BRs and that the EAT regulates cardiocirculatory autonomic nervous system activity by influencing the BRs. Normalization of cardiocirculatory autonomic activity may improve AD in chronic epipharyngitis. The evaluation by AS test load in this study showed no significant difference between the improvement group and the invariant group. It is possible that there was a problem in the classification method of the improvement group and the invariant group. Problems in classification by type of subjective symptoms, classification by severity of subjective symptoms, and how to evaluate the degree of improvement were considered. In the future, it will be necessary to improve the problems in this study and to collect more cases for further investigation.

The abstract of this paper was presented at the 122nd Annual Meeting of the Japanese Otorhinolaryngological Society (May 12-15, 2021, Kyoto, Japan).

## Conclusions

As a temporal effect of EAT stimulation, the improvement group showed significant suppression of parasympathetic activity. Evaluation by the AS test load showed no significant difference between the improvement group and the invariant group, and the interaction effect of EAT on BR was not clear. Although no significant difference was found, the improvement group showed a suppression of parasympathetic activity and a stimulation of sympathetic activity compared to the invariant group. Blood pressure in the improvement group was found to decrease, suggesting that EAT may exert its therapeutic effect on cardiocirculatory autonomic symptoms through a temporal effect on BR.
